# aKmerBroom: Ancient oral DNA decontamination using Bloom filters on k-mer sets

**DOI:** 10.1016/j.isci.2023.108057

**Published:** 2023-09-29

**Authors:** Camila Duitama González, Samarth Rangavittal, Riccardo Vicedomini, Rayan Chikhi, Hugues Richard

**Affiliations:** 1 Institut Pasteur, 75015 Paris, France; 2 MF1 - Genome Competence Center, Robert Koch Institute, 13353 Berlin, Germany; 3 Sorbonne Université, Université Paris Cité, 75005 Paris, France; 4 Independent researcher

**Keywords:** Microbial genomics, Biocomputational method, Sequence analysis, Paleogenetics

## Abstract

Dental calculus samples are modeled as a mixture of DNA coming from dental plaque and contaminants. Current computational decontamination methods such as Recentrifuge and DeconSeq require either a reference database or sequenced negative controls, and therefore have limited use cases. We present a reference-free decontamination tool tailored for the removal of contaminant DNA of ancient oral sample called aKmerBroom. Our tool builds a Bloom filter of known ancient and modern oral k-mers, then scans an input set of ancient metagenomic reads using multiple passes to iteratively retain reads likely to be of oral origin. On synthetic data, aKmerBroom achieves over 89.53% sensitivity and 94.00% specificity. On real datasets, aKmerBroom shows higher read retainment (+60% on average) than other methods. We anticipate aKmerBroom will be a valuable tool for the processing of ancient oral samples as it will prevent contaminated datasets from being completely discarded in downstream analyses.

## Highlights

• Current decontamination methods of ancient oral metagenomic samples are suboptimal
• aKmerBroom is a new tool for decontamination of ancient dental calculus samples
• aKmerBroom is a reference-free tool that builds a Bloom filter of known oral k-mers
• aKmerBroom achieves high sensitivity and specificity on synthetic and real datasets

## Graphical abstract

**Figure:**
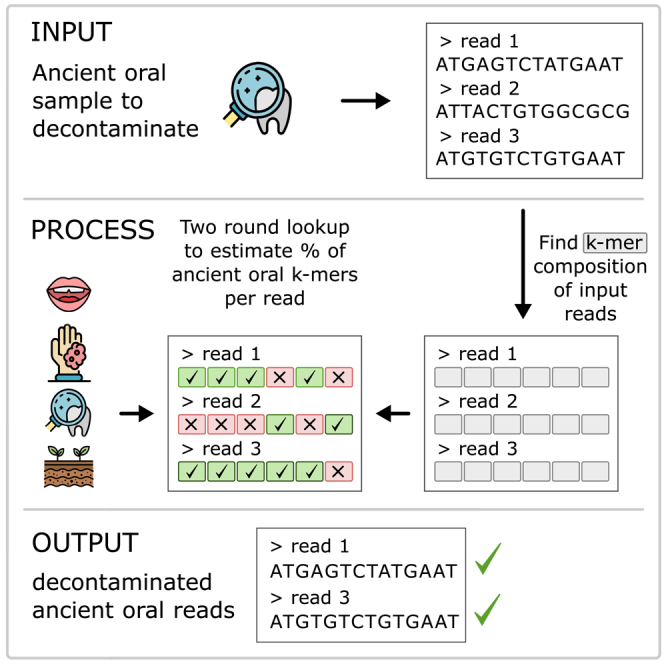

## Introduction

Ancient human dental calculus is a rich source of information on the oral microbial community that allows the study of the oral microbiome evolution, human oral health and diet.^1^ It is one of the most relevant sources of isolation in the field of paleometagenomics as it is one of the richest sources of ancient DNA (aDNA) and a crucial reservoir of ancient microbial communities.^2^^,^^3^ However, such samples are highly susceptible to contamination from environmental sources, which can drastically alter the microbial composition and lead to erroneous conclusions after downstream analyses.^4^ Several studies have shown that contaminant DNA and cross-contamination can confound metagenomic studies, and low microbial biomass samples are particularly vulnerable to contamination.^5^^,^^6^^,^^7^ Under these circumstances, contamination estimation and removal are fundamental to avoid the aforementioned risks.^5^^,^^8^ In this work, we focus on the removal of contaminated sequences in ancient oral metagenomes.

There are standardized laboratory protocols for the decontamination of aDNA samples, guidelines to minimize contamination,^1^^,^^9^ as well as bioinformatics pipelines for aDNA authentication.^10^^,^^11^^,^^12^ For human aDNA, authentication requires to single out genuine ancient human DNA (normally based on characteristic damage patterns and endogenous content^13^), as contamination from field scientists can occur at any stage, from the excavation to the DNA library preparation.^10^ In paleometagenomics, it is often very difficult to resample from rare and precious biomaterials.^4^^,^^13^ This makes decontamination procedures crucial to ensure the best use of available genetic information, while maintaining low levels of contamination. Apart from wet-lab based methods for contamination control (e.g., experimental methods), tools such as DeconSeq^14^ or Recentrifuge^15^ have digital procedures to remove genomics sequences that correspond to negative control samples. They are however not tailored for aDNA and require either a database of negative controls or an index of reference genomes to distinguish the contamination that should be removed from endogenous material. Moreover, due to the nature of the biosamples processed in aDNA studies, researchers face the challenge of having small sample sizes, a typical feature of the ancient metagenomics field that often leads to underpowered studies.^13^

In principle one could also perform read decontamination by read mapping, for instance, to a database of oral microbiota reference genomes while keeping only the reads that align with sufficient identity. However such a reference database does not exist, and the diversity of ancient oral microbiomes is not yet well characterized.^16^ Alternatively, one could decontaminate a sample by taking out reads that align to a database of known contaminant reference genomes, such as soil and skin microbes—however no such database exists and is unlikely to be created given the extensive diversity of these environments.^17^ Hence, mapping-based approaches are currently unsuitable for the decontamination of ancient metagenomes, and one must rely on alternative approaches, such as the one presented here.

Terabytes of ancient metagenomic data exist in public repositories, and also petabytes of metagenome data have been produced over diverse environments. As an attempt to globally make this huge amount of data accessible, bioinformaticians have developed efficient algorithmic methods to aggregate substrings of genomic sequences of length k, called k-mers, within these collections. Using tools such as kmtricks^18^ one can rapidly construct a matrix of k-mers from large metagenomic collections, allowing to jointly analyze all k-mers present within hundreds to thousands of metagenomic samples. However, such aggregation of k-mer information over hundreds of metagenomes has never been applied to the problem of decontaminating aDNA reads yet.

We developed aKmerBroom, the first method able to decontaminate ancient oral DNA samples without the need for a control sample nor an extensive set of reference genomes. Our method leverages the wealth of existing ancient oral metagenomes by constructing a database of ancient oral k-mers used to capture reads likely to be of ancient oral origin. In essence, aKmerBroom projects the k-mers from an input sample onto a database of reference k-mers and then selects the reads with enough coverage. Technically aKmerBroom performs a two-step lookup in a Bloom filter (BF) of oral k-mers, and then in a set of ‘anchor” reads. We evaluate aKmerBroom on three distinct synthetic datasets and on two real datasets and compare the results with current computational methods for contamination removal. Given its high sensitivity and specificity, aKmerBroom is expected to be a useful tool for decontaminating ancient oral samples.

### Related work

The advent of large scale metagenomic projects such as the Human Microbiome Project,^19^^,^^20^ the Earth Microbiome Project,^21^ Tara Ocean,^22^ or MetaSub^23^ among others, has generated large collections of modern metagenomic sequencing data that has fundamentally changed the study of microbial ecology. Other studies, at a smaller scale, still produced considerable amounts of ancient metagenomic data (approximately 1,000 sequencing runs).^24^ All these sequencing efforts came with increasing amounts of experimental noise, e.g., contamination which plagues both modern and ancient metagenomics. By contamination, we refer here to the observation of sequenced reads in a sample coming from microorganisms that were not originally part of that sample of interest.^25^

There are several computational pipelines tailored for the detection of contaminating DNA after sequencing has been performed.^26^ Yet we are not aware of tools developed specifically for contamination removal in ancient oral DNA at the read level, despite this sample type being one of the most prevalent source of aDNA.

DeconSeq,^14^ published in 2011, is a method built to detect and identify contamination in microbial metagenomes.^27^ It takes as input a set of reads, and compares it against a reference database using a modified version of the BWA-SW algorithm.^28^ DeconSeq uses different databases depending on whether the user wants to remove or retain reads. None of the databases were built for ancient oral metagenomic decontamination. The user might create their own ancient index for contaminant screening but this requires having a reference of control samples and increases the running time for contamination removal.

A previous study suggested that the use of negative controls alone is insufficient to inform researchers of measures to minimize contaminants.^6^ Tools such as decontam^29^ use pre-sequenced quantification data such as Operational Taxonomic Unit (OTU) tables, and remove contaminant taxa from such tables but do not remove contaminants at the read level. On the other hand, microDecon^30^ uses proportions of contaminant OTUs from blank samples (negative sequencing controls processed with the same DNA/PCR amplification kits as the real samples, sequenced on the same run^31^), and also adjust read counts in OTU tables but does not decontaminate the reads themselves. As they are control-based those methods do not account for cross-contamination.

Finally another tool, Recentrifuge,^15^ identifies cross-contaminations, i.e., DNA exchange between samples within one same study that can create batch effects.^29^^,^^32^ It is based on Centrifuge,^33^ a taxonomic classifier that uses the Burrows-Wheeler Transform (BWT) and an FM-index to store and index a reference database. Recentrifuge reads the score given to the reads by a taxonomic classification software (such as Centrifuge), and uses this information to calculate an average confidence level for each taxon in the taxonomic tree associated with the sample analyzed. Tools of this kind rely on sequencing blank samples (controls) to determine baseline contaminant levels of microbes.

**Figure 1:**
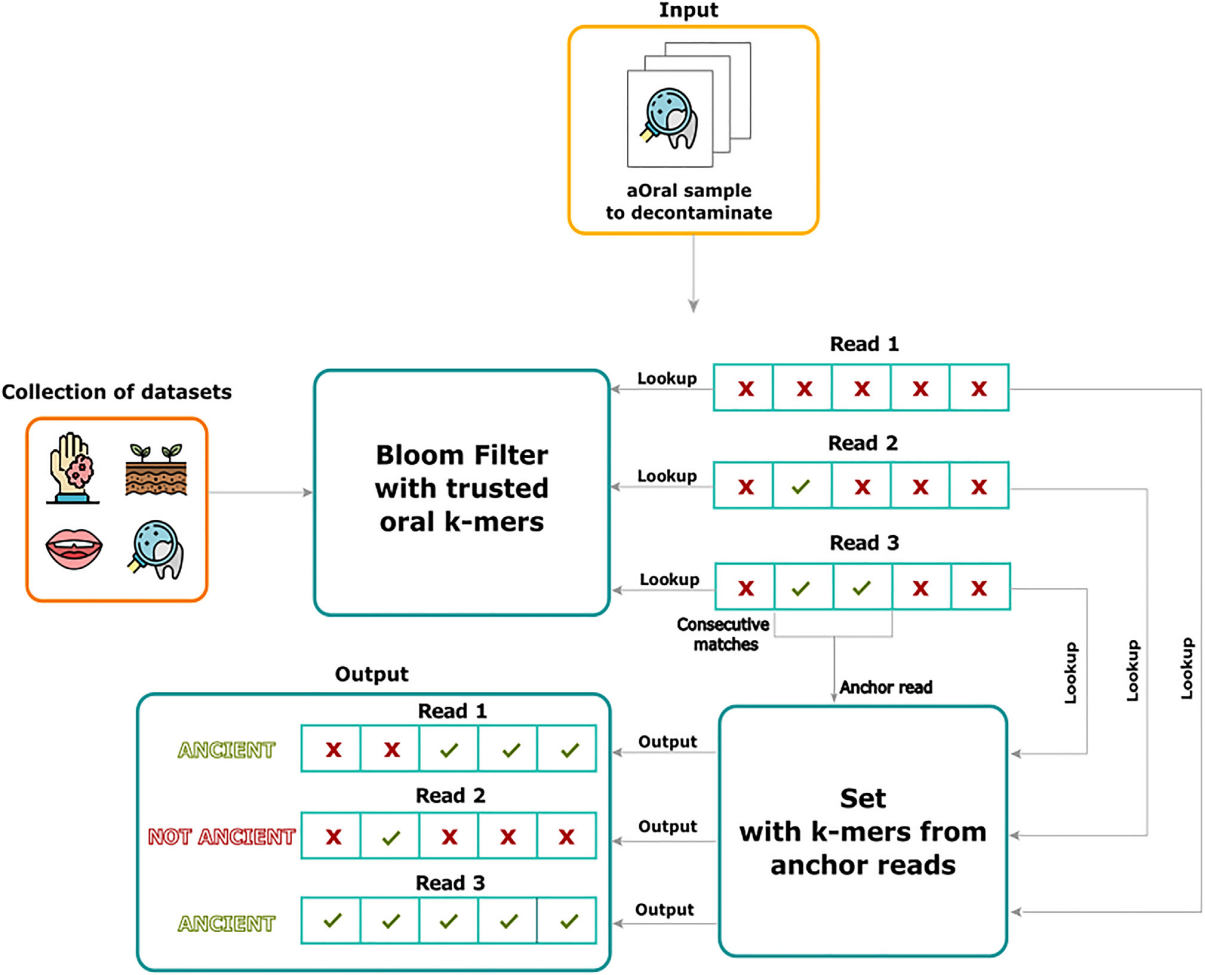
aKmerBroom pipeline First, an offline step is performed: a collection of samples representative from diverse sources is used to create a trusted set of oral k-mers. The trusted collection indexes k-mers that appear exclusively in modern and ancient oral samples, but not other samples from contaminant sources (see panel on the left called Collection of datasets). Then this set of oral k-mers is used to decontaminate an input set of reads. The algorithm proceeds by looking up each read k-mer inside the Bloom Filter of trusted oral k-mers, and marking positions of matches. Reads having at least two consecutive matches to the Bloom Filter get passed to the construction of a set containing all k-mers from such reads. Finally, the same input reads are scanned again using the aforementioned set, and reads having a proportion of k-mer matches over a certain threshold are reported to be of ancient oral origin.

To summarize, existing methods are not tailored for the decontamination of ancient oral metagenomic projects, as they are reference-based, and have not been recently updated to scale up to modern dataset sizes (such as DeconSeq) or rely on the sequencing of controls which has limited uses (such as microDecon or Recentrifuge). To remedy this, we propose aKmerBroom as a fast, reference-free and precise tool for the decontamination of ancient dental calculus samples (see Figure 1 for a brief explanation of our method).

## Results

### Datasets

To evaluate aKmerBroom in a controlled setting with known levels of contamination, we constructed three distinct synthetic datasets, corresponding to various scenarios. The Synthetic 1 and Synthetic 2 datasets correspond to the case where all or part of the reads observed in the target sample are from samples used to construct the trusted oral k-mer set, and are thus easier to decontaminate. The Synthetic 3 dataset corresponds to a case where we observe a completely new and unseen sample. Each dataset is built with an equal number of reads belonging to each of the three categories aOral, Sediment/Soil, and Skin, in a 1/3: 1/3: 1/3 proportion. We also used two real datasets from an ancient oral microbiome study. Table 1 presents the datasets.

• **Synthetic 1**: We collected 2 million reads from a source soil dataset, 2 million reads from an aOral sample and 2 million reads from a skin sample. All these source samples were present in the k-mer matrix used to create the trusted k-mers set, hence this is a best-case scenario for decontamination.
• **Synthetic 2**: A second dataset was built by sampling 2 million reads from an external aOral sample that was **not** used to create the trusted k-mers set. We added the 2 million reads from the skin and sediment/soil datasets used for Synthetic 1. Hence this dataset is a semi-artificial best-case scenario.
• **Synthetic 3**: A third and final synthetic dataset was built by sub-sampling reads from aOral, soil and skin datasets that were **not** used to create the trusted k-mers set. For the construction of this dataset, 2 million reads were sub-sampled from am aOral sample, a sediment/soil sample, and a skin sample, respectively.
• **Real data**: Lastly, we evaluated decontamination on two real datasets: First, an aOral sample (accession SRA:ERR5670971), isolated from Trentino-South Tyrol, Italy, and dating from the Early Middle Ages (400–1000 CE). Second, a real dataset (accession SRA:ERR5670966) isolated from Venosta Valley and dating from the Early Middle Ages too.^16^ None of these datasets were used to create the set trusted k-mers. A negative control sequenced and published in the same study was used to run Recentrifuge and DeconSeq, but not aKmerBroom.

**Table 1:** Composition of synthetic and real datasets

Dataset	aOral source	Skin source	Sediment/Soil source	nReads (M)	Used to build BF
Synthetic 1	SRA:SRR12462946	SRA:SRR1620017	SRA:ERR671934	6	Entirely
Synthetic 2	SRA:SRR13355797	SRA:SRR1620017	SRA:ERR671934	6	Partially
Synthetic 3	SRA:ERR3003655	SRA:SRR11426385	SRA:ERR3458820	6	No
Real 1	SRA:ERR5670971	SRA:ERR5670972		64.6	No
Real 2	SRA:ERR5670966	SRA:ERR5670972		47.8	No

For the real dataset, the accession reported for the aOral source corresponds to the sample likely to contain ancient oral microbes, to be decontaminated. The sample reported in the real datasets Sediment/Soil source is a negative control.

### Evaluation method

As we know the exact number of reads coming from the aOral sample in each of the three balanced synthetic datasets, we estimated specificity and sensitivity by calculating the true positive rate (TPR) and false positive rate (FPR). We considered as true ancient oral any read recovered by aKmerBroom coming from the aOral samples, and considered as false aOral the reads coming from the soil/skin samples. On the other hand, as we do not know the true number of contaminant reads for the real dataset, we evaluated performance by measuring read retainment, that is the percentage of original reads that were kept after contamination removal.

Competing decontamination methods such as Recentrifuge and DeconSeq were only evaluated on real data since they require negative controls or reference databases which were not available for our 3 synthetic samples. Recentrifuge relies on Centrifuge^33^ for taxonomic classification of an input set of reads. We used Centrifuge version 1.0.4-beta on a pre-made index of RefSeq bacteria, archaeal, viral, and human sequences.^34^ DeconSeq standalone version 0.4.3 was used for performance comparison against aKmerBroom on real data. To evaluate the composition of the samples before and after decontamination, we performed a contamination assessment with SourceTracker and using as sources our reference database of 360 metagenomic samples.

### Evaluation of decontamination on synthetic data

**Figure 2:**
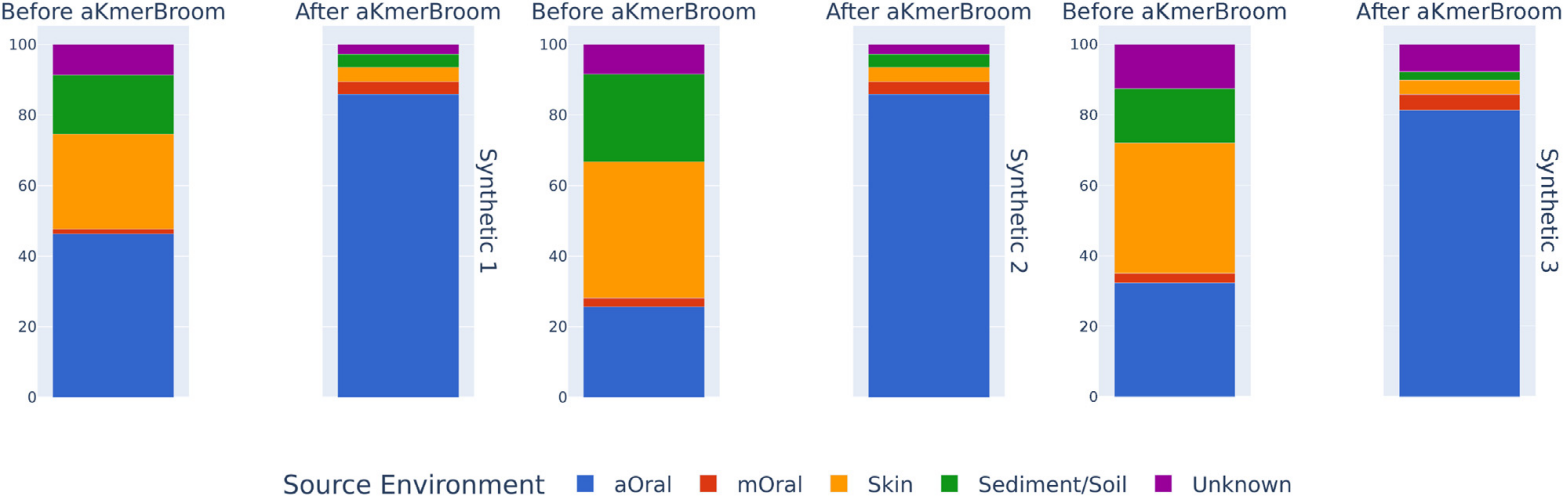
aKmerBroom pipeline aKmerBroom performance on synthetic data as evaluated by SourceTracker. We evaluated the source environment composition of each synthetic sample before and after decontamination with aKmerBroom using SourceTracker and our reference collection of 360 metagenomic samples as sources.

Table 2 reports that aKmerBroom has excellent performance (≥93% sensitivity and specificity) on synthetic datasets 1 and 2. Synthetic dataset 3 was built by sub-sampling from datasets that were not seen during construction of the trusted k-mers set, hence it is a more realistic case. Here aKmerBroom still performs remarkably well with 89.57% sensitivity and 94.00% specificity, albeit shows lower sensitivity than in the first two synthetic datasets. Contamination assessment analyses using SourceTracker (Figure 2) show that after decontamination with aKmerBroom the final oral composition is above 80% in the three synthetic datasets. This proves that also with alternative metrics to sensitivity and specificity, such as source environment proportions given by MST analyses, our method performs contamination removal effectively.

**Table 2:** Performance of aKmerBroom on synthetic samples

Dataset	Sensitivity (%)	Specificity (%)
Synthetic 1	97.85	98.00
Synthetic 2	90.84	97.96
Synthetic 3	89.53	94.00

Sensitivity is the percentage of aOral reads that were successfully retained. Specificity is the percentage of non-aOral reads that were successfully removed.

### Evaluation of decontamination on real data

When evaluating aKmerBroom on real data, we measured performance with read retainment and compared results with two competing methods: DeconSeq and Recentrifuge. We took two aOral metagenomic samples isolated from the dental calculus microbiome of two people buried in Italy in the Early Middle Ages (400–1000 CE).^16^ Researchers of this study also published a sequenced blank, which we used to create the database for contaminant screening and run DeconSeq with. That same blank was used as negative control when running Recentrifuge, in order to have a reference of taxa that needs to be removed.

The group that collected, sequenced, and published those real datasets performed several aDNA authentication analyses to prove their samples were representative of the ancient calculus microbiome. Among others, they ran SourceTracker^35^ on the aOral samples and showed that the reads stemming from a known source were predominantly coming from modern calculus and plaque,^16^ i.e., oral sources. Thus we expect a highly reliable ancient oral content in the real sample evaluated, and a low level of contamination. For this reason we used read retainment and confirmed that aKmerBroom preserves most of the reads of the original aOral sample (92.56%), whereas Recentrifuge and DeconSeq remove most of the sequences (see Table 3).

**Table 3:** Decontamination performance on two real datasets

Dataset	Run accession	Method	Reads retained (%)	O.C. before (%)	O.C. after(%)
Real dataset 1	ERR5670971	DeconSeq	8.16		84.00
		Recentrifuge	46.64		23.78
		aKmerBroom	92.56		75.11
Real dataset 2	ERR5670966	DeconSeq	22.63		75.89
		Recentrifuge	40.00		51.19
		aKmerBroom	87.42		83.09

Four methods were run to decontaminate two samples. For DeconSeq and Recentrifuge, corresponding negative controls were provided as input too. The nReads column shows the total number of reads in case and control samples. The column O.C. (Oral Content) refers to the proportion of oral source environment in the sample *after* and *before* contamination removal with each of the methods, as estimated by SourceTracker.

**Figure 3:**
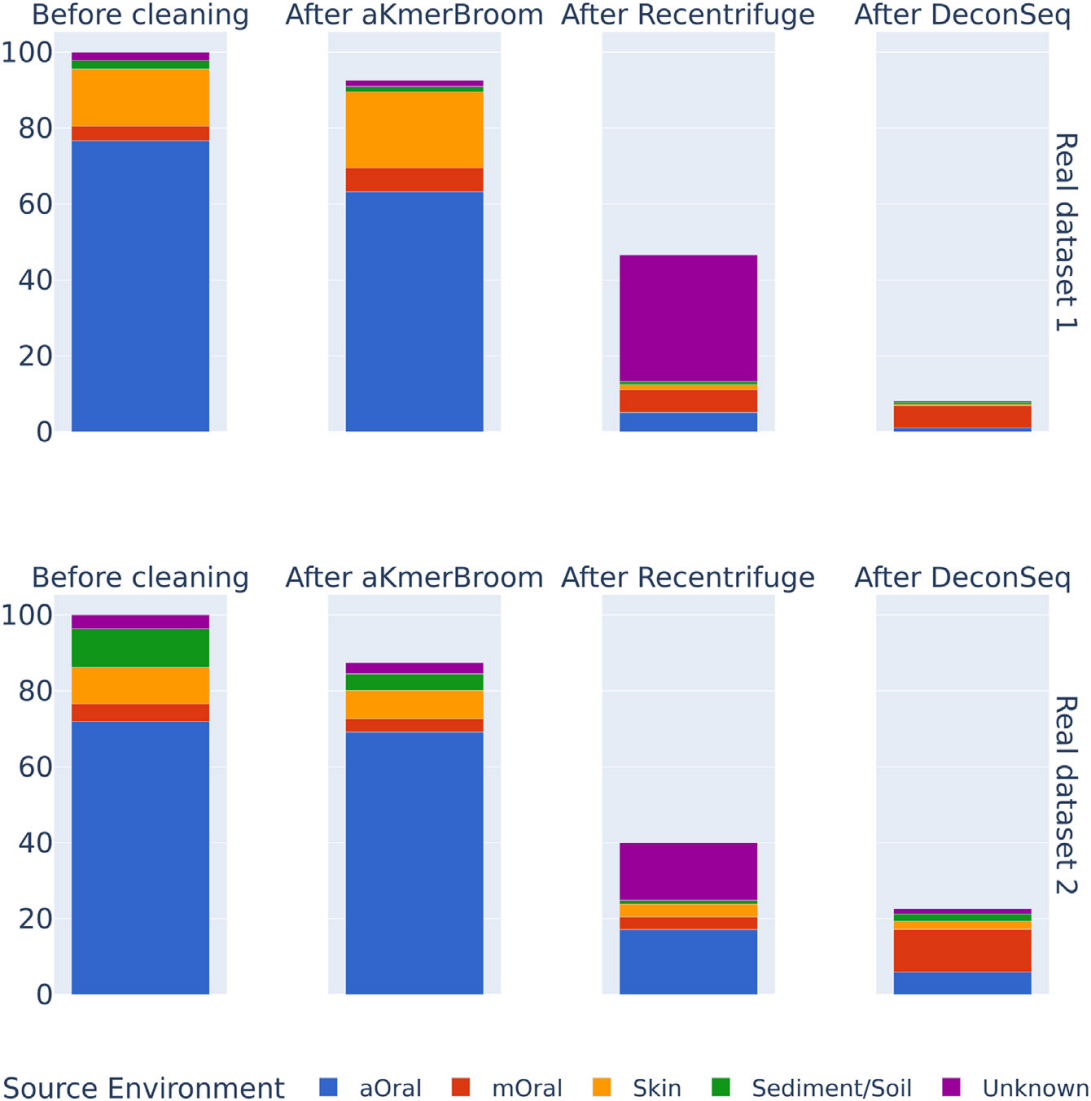
aKmerBroom performance on synthetic data as evaluated by SourceTracker We evaluated the source environment composition of each synthetic sample before and after decontamination with aKmerBroom using SourceTracker and our reference collection of 360 metagenomic samples as sources.

We additionally performed an evaluation of the real samples of ancient oral origin, by running mSourceTracker on each of the samples and against a set of sources represented with an OTU table built from our reference collection of 360 metagenomic samples (sources: ancient oral [aOral], modern oral [mOral], Sediment/Soil and Skin) (further details on the OTU table construction and taxonomic classifier used are detailed in the Supplementary material of decOM^36^). Results are presented in Figure 3.

### Computational performance

Using the pre-constructed BF of oral k-mers, aKmerBroom has a runtime of around 1 h for a dataset with fewer than 10 million reads, while using approximately 10 Gb of memory. Beyond this input size, the run time and memory requirement scales linearly with the number of unique anchor k-mers in the input reads. Note that if a new BF has to be constructed from scratch, this one-time step would take around 6 h for a file of 1 billion k-mers.

Leaving out the time to build the BF or index the control/reference database and evaluating running time on Real dataset 2, a FASTA file of almost 48 million reads, DeconSeq took around 1 day to run, aKmerBroom took around 4 h, and Recentrifuge 2 h. Both DeconSeq and Recentrifuge were run using 2 Gb of memory.

## Discussion

Decontaminating ancient oral metagenomes is a challenging computational problem, currently poorly performed using off-the-shelf tools. This work highlights that current ancient metagenomic studies are hindered by suboptimal decontamination methods. We propose aKmerBroom, a tool for contamination removal of ancient oral datasets using a BF constructed on a set of trusted oral k-mers, using a large collection of metagenomes.

We evaluated aKmerBroom with three distinct synthetic metagenomic datasets subsampling from sample sources that were fully, partially and non-included in the construction of the BF, and obtained 97.85%, 93.39%, and 89.53% sensitivity and 98.00%, 97.96%, and 94.00% specificity (respectively). We further measured aKmerBroom performance on two real samples and quantified the percentage of ancient oral sample preserved. aKmerBroom effectively preserves most of the original sample, and removes contaminant reads as estimated by SourceTracker, whereas other methods (Recentrifuge, DeconSeq) discard over 53% of the sequences and remove true ancient oral content, also as estimated by SourceTracker.

k-mer-based methods such as aKmerBroom are relevant to modern day DNA analyses because they are reference-free (e.g., they do not require a database of reference genomes) and they make use of the large corpus of genomic information that has been gathered over the years. Since we use a k-mer-based consensus of samples to decide what to keep, but we do not decide which species specifically are present/absent in the input sample, our method does not suffer from biases coming from using OTU tables or reference databases. Others have reported using k-mers to assess contamination in human whole-genome samples by doing meta analyses across different datasets.^37^ Thus a trend emerges on using stored genetic information to tackle the problem of contamination assessment and removal, instead of making it a matter that is unique to each study.

As it simplified the implementation, the second round of lookups was performed using an exact membership data structure (set). Yet as a future step, performance improvements can be made to reduce the memory requirement for large input files with a significant proportion of ancient reads. For example, this second round could also be implemented using a BF. This way the memory required can then be independent of the size of the input reads dataset.

The fact that ancient metagenomic samples are rare and have low biomass of ancient remains often translates into underpowered aDNA studies. As more and more ancient metagenomic projects are published, tools such as aKmerBroom emerge as a novel and efficient way of incorporating data from previous studies by concisely storing information in the form of a BF. Unlike other methods, aKmerBroom represents the variety of ancient oral metagenomic material across several BioProjects, while not making specific assumptions about the microbial species that should be expected or ignored. It mitigates the effect of small sample size (as the output of several metagenomics studies are put together to construct the BF) while still making computationally manageable analyses.

**Figure 4:**
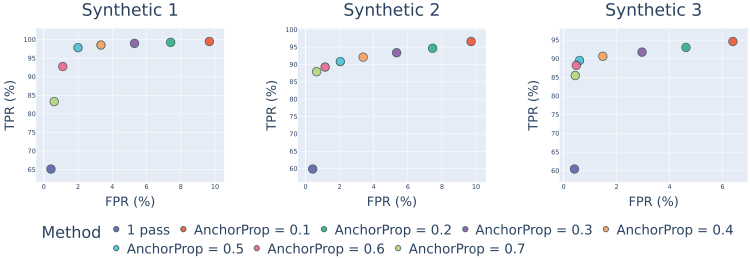
ROC (Receiver Operating Characteristic) curve for selection of anchor proportion threshold We optimised the threshold to decide if a read would be classified as ancient or not by running aKmerBroom with different values of the parameter τ (the proportion of k-mers found in the anchor k-mers set) and evaluating every run with sample Synthetic 1. As seen on the left panel, the value of τ that has the best trade-off between a high True Positive Rate (TRP) and a low False Positive Rate (FPR) is 0.5. We additionally evaluated an earlier version of aKmerBroom that did not include matches against anchor reads and performed only one lookup step in the BF, represented with the blue marker called 1 Pass. Results for samples Synthetic 2 and Synthetic 3 are presented in the middle and right panel respectively.

aKmerBroom brings usability improvements to decontamination methods. Prior to it, users had to make decisions on how to properly carry out analyses. For instance, in the case of Recentrifuge, one needs to estimate whether to run the taxonomic classifier Centrifuge with default or modified parameters, selecting for a pre-made index or building an index with the criteria of the user, which is equivalent to curating a database that ideally would be tailored for ancient oral decontamination. In the case of DeconSeq, users have to select either a “retain” and/or a “remove” database, plus other alignment options that affect BWA-SW results. All these decisions are required even for non-expert users, and they have not been properly benchmarked for aDNA analysis, ultimately leading to sub-optimal results. Although it is out of the scope of this paper to do parameter optimization on all methods to tailor them for ancient oral datasets, we introduce here a method that overcomes much of the parameter selection and database creation burdens that exist in the other decontamination tools. Furthermore, aKmerBroom's main parameter is τ, the anchor proportion threshold, that is used to decide if a read is ancient or not. This parameter has already been optimized for users of our method (see Figure 4 and STAR Methods section for more details).

Some researchers have often emphasized the importance of including negative controls to understand background contamination.^38^ While others have focused on implementing the strategy of identifying a “contaminome” profile or list of possible contaminant taxa, to then remove it from the studied sample.^31^^,^^37^ The latter, however, rises doubts on whether it can really take into account the possibility that contaminants may come from other samples within the same study.^25^ One interesting future work would be to specifically test for this between-sample contamination using aKmerBroom and compare performance with methods such as Recentrifuge that are tailored specifically to tackle cross-contamination.

### Limitations of the study

We rely on the metadata of each metagenomic sample to assign a true label (i.e., environment type), however, there is no ground truth as to what is the true proportion of aOral, mOral, Sediment/Soil or skin content in any of them. Overall, one of the biggest challenges in the field of paleometagenomics is that there is not a straightforward (taxonomic) characterization of an ancient oral metagenome, modern oral metagenome or a contaminant metagenome. Following that line of thought, we acknowledge that our creation of a set of trusted oral k-mers is only an approximation to what a “clean” ancient oral set of k-mers might look like, but there is no way to know for sure that this set of k-mers *only* contains ancient oral DNA. On the other hand, we allow users to input their own set of k-mers for the construction of their own BF, with the hope that experts in the field might be able to come up with their own trusted set of oral k-mers validated by their biological understanding of the problem.

We acknowledge read retainment (percentage of original reads that were kept after contamination removal) is a proxy for how clean a sample is after decontamination, but there might be additional analyses that can be done to authenticate the true content of an ancient oral sample after using aKmerBroom, that take into account additional biological information such as deamination or fragmentation patterns.

As most of the k-mers that were used for the construction of the trusted set of oral k-mers come from Illumina HiSeq reads, we know that a limitation of aKmerBroom is that it may not show the same performance with higher error rates.

Despite having effectively used a two-round lookup using a BF and a set to construct aKmerBroom and decontaminate synthetic and real ancient oral data, there might be more memory-efficient data structures to effectively perform the same task that are worth exploring in the future.

## STAR★Methods

### Key resources table

**Table:** 

REAGENT or RESOURCE	SOURCE	IDENTIFIER
**Biological samples**		

aOral source for Synthetic 1	(Jacobson et al.^39^)	SRA:SRR12462946
Skin source for Synthetic 1	(Turnbaugh et al.^40^) HMP	SRA:SRR1620017
Sediment/Soil source for Synthetic 1	(Bissett et al.^41^) BASE Project	SRA:ERR671934
aOral source for Synthetic 2	(Farrer et al.^4^)	SRA:SRR13355797
aOral source for Synthetic 3	(Velsko et al.^42^)	SRA:ERR3003655
Skin source for Synthetic 3	(Kim et al.^43^)	SRA:SRR11426385
Sediment/Soil source for Synthetic 3	(Cribdon et al.^44^)	SRA:ERR3458820
Real 1 aOral sample	(Farrer et al.^4^)	SRA:ERR5670971
Negative control for real samples	(Farrer et al.^4^)	SRA:ERR5670972
Real 2 aOral sample	(Farrer et al.^4^)	SRA:ERR5670966

**Deposited data**		

test_1	this paper	https://doi.org/10.5281/zenodo.7590899
test_2	this paper	https://doi.org/10.5281/zenodo.7590899
test_3	this paper	https://doi.org/10.5281/zenodo.7590899

**Software and algorithms**		

aKmerBroom	this paper	https://zenodo.org/record/7156306

### Resource availability

#### Lead contact

Further information and requests for resources should be directed to and will be fulfilled by the lead contact, Camila Duitama (cduitama@pasteur.fr).

#### Materials availability

This study did not generate new unique reagents.

### Method details

We have developed aKmerBroom, the first method able to perform read-level decontamination on ancient oral metagenomes. As an input to aKmerBroom, the user provides a set of reads to be decontaminated. aKmerBroom then scans the input reads against a set of oral k-mers, using two passes to iteratively retain reads likely to be of ancient origin.

The main steps are described below, and a high-level summary is provided here. First a set of high quality oral k-mers is determined from a database of ancient and modern oral samples as well as environmental samples. Then, a Bloom Filter is constructed to represent this set approximately in memory. The tool then scans input reads and retains those that have at least 2 consecutive k-mer matches against the filter. Those reads enable us to enrich the set of ancient k-mers by incorporating new putative ancient k-mers. We refer to those reads as “anchor reads”. We then perform another pass over the input reads and identify matching reads against this new subset of k-mers. Reads are finally classified as ancient when ≥50% of their k-mers match the set of k-mers generated from anchor reads.

#### Creating a set of trusted oral k-mers

To construct a set of trusted oral k-mers for aKmerBroom, we use a resource from decOM,^36^ a method for contamination assessment of ancient oral metagenomic samples. decOM constructs a k-mer matrix from 360 metagenomic samples covering a wide range of environments around the world, labelled as ancient oral (aOral), modern oral (mOral), and their possible contaminants (Sediment/Soil and Skin samples). Sample accession numbers are provided in the supplementary material of the decOM publication.^36^ The decOM matrix is built over distinct k-mers of size 31, filtered by retaining k-mers that were present in at least 3 samples in the collection and by removing all k-mers seen only once in a sample, which were likely to be sequencing errors. In order to reduce memory usage, we start by subsampling 10% of the k-mers present in the decOM matrix, we select a set of a high quality oral k-mers by filtering each k-mer that satisfies all of the following conditions:

• Present in any of the aOral samples or,
• Present in any of the mOral samples and,
• Absent in all Skin samples and,
• Absent in all Soil samples.

In a boolean formula the conditions could be read as (inAOral or inMOral) and not(inSkin or inSoilSediment).

We obtain over 1.5 billion k-mers, which corresponds to roughly 25% of the subsampled set of k-mers matrix (2.5% of whole set of k-mers of the decOM matrix of sources). These k-mers are referred to as *trusted oral* k*-mers* .

We would like to emphasise that our method is reference-free as it does not require a database or index of reference genomes, however, in the construction of the Bloom Filter, there must be a reference of k-mers considered to be of ancient oral origin. We have defined this set after the conditions previously explained, but the user might come up with their own input set of k-mers too.

#### Constructing a bloom filter from oral k-mers

A Bloom Filter is a space-efficient probabilistic data structure that enables to query the membership of an element within a set, with false positives but no false negatives.^45^ As a preprocessing step, aKmerBroom constructs a Bloom Filter (BF) from a set of k-mers (using pybloomfiltermmap^46^). In aKmerBroom, the user may provide their own set of k-mers, or alternatively use the pre-constructed table of trusted oral k-mers provided with the software and constructed as described in the previous section (see Zenodo file^47^). In the upcoming section, we discuss how we mitigate the issue of false positives.

#### Pass 1: Finding anchor reads

In the first pass, aKmerBroom scans each read and looks for k-mer matches in the Bloom Filter. If two consecutive k-mer matches are found, a read is marked as an “anchor” read. These anchors will be used in the next pass to identify reads with ancient origin. Note that requiring only two consecutive k-mer matches has the advantage of being permissive, while also avoiding cases when a single false positive match might result in the read being falsely included as an anchor read.

#### Pass 2: Identifying ancient reads

All anchor reads from the first pass are k-merized and stored into a new anchor k-mer set. The full input dataset is scanned again, and reads having a proportion ≥50% of k-mers present in this new anchor k-mer set are retained as likely to be of ancient origin. Note that non-anchor reads may be retained, as some will satisfy this criteria. The final output of aKmerBroom consists of the set of retained reads.

#### Parameter selection

The aKmerBroom method relies on one main parameter: the anchor proportion threshold τ. In addition, the Bloom Filter implementation requires two other parameters: the capacity and the error rate. There is a trade-off between these two parameters: adding less than *capacity* items ensures that the Bloom Filter will have an error rate less than *error rate*.^46^ In aKmerBroom, we set the error rate to be 0.001, and set the Bloom Filter capacity to be at least as large as the number of trusted k-mers to be stored. By default, we set it to be 2 billion so that it is larger than the 1.5 billion pre-computed trusted k-mers. One could increase the capacity of the Bloom Filter (or decrease the tolerated error rate), but that would result in a larger Bloom Filter and therefore increase memory requirements. To determine an appropriate value for the anchor k-mer proportion threshold τ, we performed a standard grid search from 10% to 90% over Synthetic dataset 1 (see results). As shown in Figure 4, we chose a threshold of 50% because it gives us a suitable trade-off between having a high true positive rate (greater than 85%) while also having a low false positive rate (less than 5%). However, the user can also set τ according to their desired sensitivity/specificity trade-off.

Originally we had tried out only one pass over the initial Bloom Filter built from the set of high quality oral k-mers, and we further improved the results by implementing a second pass based on matches against anchor reads identified from the first pass. Results for this one-step version of aKmerBroom are also shown in Figure 4 (“1 pass”). Notice that the method with only one pass performs worse than any of the thresholded two-pass methods.

#### Output description

aKmerBroom outputs an annotated FASTQ file with 4 fields in the record header:

• **SeqId**: sequence identifier
• **ReadLen**: length of the read
• **isConsecutiveMatchFound**: a binary variable to indicate if 2 consecutive k-mers were found in the first lookup.
• **AnchorProportion**: percentage of k-mers that were found in the anchor k-mers set.

## Data Availability

• This paper analyses existing, publicly available data. These accession numbers for the datasets are listed in the key resources table. • Synthetic data have been deposited in a Zenodo repository https://zenodo.org/record/7590899#.Y9lQ_y9w0Us and are publicly available as of the date of publication. DOIs are listed in the key resources table. • All original code has been deposited at https://github.com/CamilaDuitama/aKmerBroom/ and is publicly available as of the date of publication. DOIs are listed in the key resources table. • Any additional information required to reanalyze the data reported in this paper is available from the lead contact upon request.
